# Connexin 43 Deficiency Confers Resistance to Immunotherapy in Lung Cancer via Inhibition of the Cyclic GMP‐AMP Synthase–Stimulator of Interferon Genes Pathway

**DOI:** 10.1111/jcmm.70211

**Published:** 2024-11-26

**Authors:** Yuan Zhang, Yu Gao, Jie Wang, Xiaoming Tan, Yusheng Liang, Weize Yu, Zihua Deng, Jingjing Zhou, Zu Ye, Guoqing Luo

**Affiliations:** ^1^ Affiliated Qingyuan Hospital, Qingyuan People's Hospital Guangzhou Medical University Qingyuan Guangdong China; ^2^ Qingyuan Maternal and Child Healthcare Hospital Qingyuan Guangdong China; ^3^ College of Pharmacy Dali University Dali Yunnan China; ^4^ Zhejiang Cancer Hospital, Hangzhou Institute of Medicine (HIM) Chinese Academy of Sciences Hangzhou Zhejiang China

**Keywords:** cGAMP, connexin 43, macrophage, STING

## Abstract

Immune checkpoint inhibitors (ICIs), especially PD‐1 inhibitors, are among the first‐line therapeutic drugs for the treatment of advanced non‐small cell lung cancer (NSCLC). However, most patients are not sensitive to PD‐1 inhibitors, and prolonged exposure can lead to acquired resistance. Thus, it is urgent to elucidate the mechanism underlying the resistance of NSCLC to ICIs. Connexin 43 (Cx43) is a gap junction (GJ) protein that is important in therapeutic efficacy to ICIs. In this study, we observed that Cx43 in murine Lewis lung carcinoma (LLC) cells mediated cyclic GMP‐AMP (cGAMP) transfer to macrophages. Knockdown of Cx43 reduced T‐cell activation, leading to decreased sensitivity of LLC cells to anti‐PD‐1 therapy. The mechanism might be that knockdown of Cx43 in LLC cells promotes macrophages differentiation into pro‐tumour M2 type (TAM), thus activating the STING pathway in macrophages. These findings indicate that downregulation of Cx43 in LLC cells leads to immunotherapy resistance by negatively regulating the cGAS–STING pathway in macrophages. Therefore, Cx43/GJ‐mediated signal transmission between lung cancer cells and macrophages provides new insights for increasing immunotherapy sensitivity in NSCLC.

## 1 Introduction

Lung cancer is the malignant tumour with the highest morbidity and mortality worldwide, with a 5‐year survival rate of only 10%–20%. According to 2022 global cancer statistics, lung cancer accounts for 12.4% of all new tumours, and the deaths caused by it account for 18.7% of all tumours [1]. Immune checkpoint inhibitor (ICI) therapy, especially programmed cell death 1/ligand 1 (PD‐1/PD‐L1) therapy, has been recommended as a first‐line treatment for non‐small cell lung cancer (NSCLC) [2]. Among them, pembrolizumab, nivolumab and atezolizumab have been approved for the treatment of patients with metastatic NSCLC. In addition, cemiplimab is approved for first‐line treatment in patients with advanced NSCLC with PD‐L1 expression levels of at least 50%, but regardless of the expression level of PD‐L1, cemiplimab combined with platinum‐based chemotherapy is needed to treat non‐squamous epithelial tissue to show efficacy in NSCLC [3]. However, recent studies have shown that the objective response rate of patients with lung cancer to PD‐1/PD‐L1 inhibitors varies between 20% and 40%, and even for PD‐L1‐high expressed patients with a PD‐L1 tumour proportion score (TPS) ≥ 50%, the response rate is only approximately 50% [4, 5, 6]. On the other hand, patients with NSCLC always develop natural or acquired resistance to ICIs. For example, clinical studies have shown that among 1201 NSCLC patients receiving PD‐(L)1 blocking therapy, more than 60% of the initial responders developed acquired resistance [7]. Therefore, increasing the sensitivity of NSCLC patients to ICIs is urgent.

PD‐1/PD‐L1 inhibitors block the interaction of PD‐1/PD‐L1 to activate T cells and generate antitumor immunity by increasing the release of cytokines such as interferon‐γ (IFN‐γ) and activating inhibited T cells [8]. Patients with poor responses to PD‐1/PD‐L1 inhibitors lack CD8^+^ T‐cell infiltration or have reduced IFN signalling in their tumour cells, which results in T‐cell dysfunction. One of the causes of T‐cell dysfunction is infiltration of tumour‐associated macrophages (TAMs) [9]. Macrophages are antigen‐presenting cells (APCs) with M1 and M2 subtypes (TAMs) that exist in dynamic equilibrium and can be transformed into each other [10, 11]. Studies have shown that TAMs can reduce the sensitivity of melanoma, rectal cancer, NSCLC and breast cancer to ICIs [12, 13]. The enrichment of TAMs and their long‐term interaction with CD8^+^ T cells makes it difficult for CD8^+^ T cells to migrate and invade tumour nests, which results in reduced T‐cell infiltration [12, 14]. Differentiation of TAMs into M1‐macrophages increases the surveillance of tumours by CD8^+^ T cells and enhances the sensitivity of tumours to PD‐1 inhibitors [15]. However, the mechanism how TAM affect NSCLC resistance to ICIs remains unclear.

Tumour‐infiltrating macrophages can secrete interferon‐activating protein (stimulator of interferon genes, STING), which promotes T lymphocytes to produce IFN signalling and exerts anti‐tumour effects [16, 17]. STING‐mediated T‐cell activation enhances the response to immunotherapy in lung cancer [18]. In KRAS‐mutated lung cancer, tumours with LKB1 mutations silence STING and reduce ICI sensitivity. However, when STING is highly expressed, KRAS and TP53 mutations are mutually exclusive. In KRAS wild‐type tumours with LKB1/TP53 comutation, STING pathway activation and immune checkpoint expression are increased [19, 20]. After STING is activated, the STING polymer recruits downstream tank‐binding kinase 1 (TBK1), which phosphorylates interferon regulatory factor 3 (IRF3). Phosphorylated IRF3 is transferred into the nucleus, where it regulates a series of downstream genes and signalling pathways, induces IFN signalling in tumour cells, and synergises with PD‐1/PD‐L1 inhibitors [21]. Studies have shown that the activation of the STING pathway is dependent on macrophages [22]. In breast cancer, rectal cancer, and melanoma, STING activation can promote the transformation of TAMs to the M1 type, increase the infiltration of CD8^+^ T cells, and increase the sensitivity to ICIs [23, 24]. Other studies have shown that STING signalling in TAMs is inhibited [25], indicating that the STING pathway in TAMs may be related to the resistance of tumour cells to ICIs. Cyclic GMP‐AMP synthase (cGAS) is a cytoplasmic DNA sensor that generates the second messenger cyclic GMP‐AMP (cGAMP) by binding to cytoplasmic dsDNA. cGAMP can bind to STING to activate downstream immune responses [26]. Studies have shown that cytoplasmic dsDNA produced by tumour cells can be delivered to macrophages, activate the cGAS–STING pathway, activate T cells, and increase the sensitivity of these cells to ICIs [27]. However, how tumour cell cGAMP is transmitted to adjacent macrophages is unclear. Kitai et al. reported that exosomes may be the transmission pathway [28], but the latest research has shown that cGAMP produced by tumour cells can be transmitted to lung macrophages via gap junctions (GJs), thereby producing an anti‐tumour immune response [28, 29].

GJs are membrane channel structures formed between cells and are composed of the special channel protein connexin (Cx). The distribution of the Cx protein is tissue specific. Immune cells and lung tissue mainly express Cx43, while the liver mainly expresses Cx32 [30]. Cx proteins and GJs play important roles in immune cell migration, antigen presentation, and immune tolerance [31]. Cx32/GJ mediates the transmission of miRNA from hepatoma cells to macrophages [32]. cGAMP from breast cancer brain metastatic cells can be transmitted to macrophages through Cx43/GJ‐composed adjacent glial cells [30], indicating that a reduction in or absence of Cx43/GJ reduces signalling between tumour cells and macrophages. Therefore, in this study, we hypothesised that Cx43/GJ in NSCLC cells mediates cGAMP transfer to macrophages and activates the cGAS–STING pathway, thus increasing anti‐PD‐1 sensitivity to NSCLC in LLC models.

## 2 Materials and Methods

### 2.1 TIMER and TIDE Analysis

The Tumor Immune Estimation Resource (TIMER2.0) platform provides integrated analyses of tumour immune infiltration alongside genetic and clinical data (http://timer.cistrome.org/) [33]. The Gene_Corr module was utilised to examine the correlation between GJA1 and CD68 expression level in lung adenocarcinoma (LUAD) and the immune gene module was utilised to examine the correlation between GJA1 and immune cell subsets, including CD8^+^ T cells, total macrophages and M1 macrophages in LUAD. The correlation results were visualised using scatter plots, with p‐values and correlation coefficients calculated via a purified Spearman rank correlation test. Additionally, the Tumor Immune Dysfunction and Exclusion (TIDE) database (http://tide.dfci.harvard.edu) integrates large‐scale histological data from CRISPR screens, non‐immunotherapy tumour samples and published immune checkpoint blockade trials [34], and was used to calculate the TIDE score and collect the OS of LUAD patients to assess the relationship between GJA1 expression and immunotherapy response.

### 2.2 Cell Lines and Cell Culture

The human embryonic kidney cell line (HEK293T), human monocyte (THP‐1), murine Lewis lung carcinoma (LLC) and human non‐small cell lung carcinoma (A549) cell lines were purchased from the American Type Culture Collection (ATCC). HEK293T LLC and A549 cells were cultured in DMEM with 4.5 g/L glucose (Corning No. 10‐013‐CV) supplemented with 10% fetal bovine serum at 37°C in an atmosphere of 5% CO_2_ in air (Thermo Fisher Scientific, SH3007103). The monocyte line THP‐1, also purchased from ATCC, was cultured in RPMI 1640 supplemented with 10% fetal bovine serum at the same temperature and atmosphere. The cell lines were tested for mycoplasma contamination.

### 2.3 RNA Interference and Stable Cell Line Generation

The shRNAs for Cx43 (14609) were obtained from Sigma Aldrich. The sequences for clones TRCN0000068473 and TRCN0000068474 were 5′‐CCCACCTTTGTGTCTTCCATA‐3′ and 5′‐GCAGATTGAAATCAAGAAGTT‐3′, respectively. The shRNA gene knockdown plasmids or the negative control were transfected via the Lipofectamine 3000 reagent (Invitrogen, No. L3000150) according to the manufacturer's instructions. After 48 h, the infected cells were selected with 500 μg/mL G418 (Gibco, 10131035) for 7 days to obtain a stable cell line. Western blotting and parachute assays were performed to ensure knockdown effiency of Cx43 and loss function of GJ. To generate A549 cells that overexpress Cx43, the target sequence NM_010288.4 was cloned and inserted into the PLVX‐puro (Tsingke, No. GZ0170498‐1) vector through EcoRI and BamHI restriction enzyme sites. Lentiviral transfection was performed in HEK293T cells via the transfection agent Lipofectamine 8000 (Beyotime, No. C0533) and the supernatant of the medium was collected and filtered through a 0.45 μm filter. We introduced lentivirus supernatants with Cx43 overexpression constructs into A549 cells, which were processed for stable selection with 2 mg/mL puromycin. Cx43 protein expression in infected A549 cells was analysed by Western blotting to confirm successful overexpression.

### 2.4 SiRNA and Cell Transfection

The siRNA for Cx43 (14609) used in this study was synthesised by TSINGKE biological technology (Beijing, China). The sequence of the siRNA used was 5′‐CUAUGUGAUGAGAAAGGAA‐3′. The working concentration of the siRNAs was 50 nM. siRNA transfection was performed by using Lipofectamine 2000 (Invitrogen, No. 11668019). The knockdown efficiency and specificity of the siRNAs were verified via Western blotting.

### 2.5 Cell Apoptosis Assay

Cell apoptosis was detected via a FITC Annexin V apoptosis detection kit (BD Biosciences, No. 556547). Briefly, cells were treated with an anti‐PD‐1 antibody with or without cGAMP, washed with cold PBS and resuspended in 1X binding buffer. Five microliters of FITC Annexin V and 5 μL of propidium iodide (PI) were added to each sample, which was subsequently incubated for 15 min at room temperature in the dark and then analysed via flow cytometry. Early (Annexin V^+^, PI^−^) and late apoptotic (Annexin V^+^, PI^+^) cells were counted as the total number of cells that underwent apoptosis.

### 2.6 Generation of Naïve Bone Marrow‐Derived Macrophages

Bone marrow cells were collected from femurs obtained from 8‐ to 10‐week‐old C57BL/6 mice. After red blood cell lysis, bone marrow cells were seeded at a density of 5 × 10^6^ cells/150 × 15 mm Petri dish and cultured at 37°C in complete Dulbecco's modified Eagle's medium (DMEM) containing 20% L929 cell‐conditioned medium, which provided macrophage colony‐stimulating factor. The macrophages were cultured at 37°C in complete DMEM and were ready for use.

### 2.7 In Vitro Coculture With T Cells

Spleens from C57BL/6 mice were harvested and filtered through a 70‐μm cell strainer to generate a single‐cell suspension. After red blood cell lysis, the splenocytes were counted and plated in complete 1640 medium supplemented with 50 μM β‐mercaptoethanol and 10 mM HEPES in 96‐well plates coated with 2.5 μg/mL anti‐CD3 (clone 145‐2C11, Biolegend) and 3 μg/mL anti‐CD28 (clone 37 N, Biolegend) antibodies. Splenocytes were activated for 24 h before being cocultured with macrophages. Macrophages were seeded with activated splenocytes at a ratio of 5:1. After coculture for another 24 or 48 h, the splenocytes were collected for flow cytometry analysis.

### 2.8 Macrophage Polarisation and Stimulation

To polarise macrophages towards an M1‐like phenotype, we stimulated Bone Marrow‐Derived Macrophages (BMDMs) with IFN‐γ (20 ng/mL, PeproTech) for 48 h. To induce an M2‐like phenotype, we treated BMDMs with IL‐4 (20 ng/mL, PeproTech) for 48 h. To stimulate macrophages with tumour cells, we cultured BMDMs with LLC, LLC‐shCx43, LLC‐siNC and LLC‐siCx43 cells pretreated with or without 2′,3′‐cGAMP (InvivoGen, tlrl‐nacga23‐02) at a ratio of 1:5 for 48 h at 37°C. BMDMs were collected for flow cytometry analysis. The same treatment was verified by coculturing A549 or Cx43‐overexpressing A549 cells with THP‐1 cells.

### 2.9 Flow Cytometry Analysis

Live single cells were stained with Fixable Viability Dye eFluor 450 (Thermo Fisher Cat# 65‐0863‐18), followed by incubation with APC/Cyanine7 anti‐mouse CD45, PerCP/Cyanine5.5‐CD8, PE/Cyanine7‐CD69, PE‐PD‐1, APC‐CD11b, FITC‐F4/80, PE/Cyanine7‐MHCII and PerCP/Cyanine5.5‐PD‐L1 (BioLegend, No. 103116, 100734, 104511, 135025, 101211, 123107, 107629, 124343) antibodies or with APC/Cyanine7 anti‐human CD45, PE/Cyanine7‐CD11b, APC‐CD206 and FITC‐CD86 (BioLegend, No. 304014, 101215, 321109, 374204) antibodies. For intracellular staining, the cells were fixed, permeabilised with a Foxp3/Transcription Factor Staining Buffer Set (eBioscience) and then stained with flurochrome‐conjugated antibodies against PE‐CD206 (BioLegend, No. 141705). For cytokine staining, the cells were first stimulated with Cell Stimulation Cocktail (eBioscience) at 37°C for 4 h and then stained with APC‐conjugated anti‐IFN‐γ (BioLegend, No. 505810). The stained cells were acquired with a BD FACSCanto II flow cytometer via BD FACSDiva software (BD Biosciences) and the generated data were processed via FlowJo software.

### 2.10 Parachute Dye‐Coupling Assay

A dye‐coupling assay was used to examine gap junction intercellular communication (GJIC) as previously described [35, 36]. Briefly, the cells were grown to confluence, and the donor cells were labelled with 5 μM calcein‐AM for 30 min at 37°C and then trypsinised and seeded onto the receiver cells at a 1:150 donor:receiver ratio. The cells were allowed to attach to the monolayer of receiver cells to form GJs for 4 h at 37°C and were then monitored under a fluorescence microscope (Olympus DP73, Tokyo, Japan). For each experimental condition, the average number of receiver cells around every donor cell was recorded as an index of GJIC.

### 2.11 Western Blotting Analysis

Western blotting was performed as previously described [15]. The following primary antibodies were used: Cx43 (Sigma Aldrich, No. C8093), STING (Cell Signalling, No. 13647), β‐actin (Sigma, No. AC‐15), GAPDH (Affinity, No. AF7021) and β‐tubulin (Sigma Aldrich, No. T4026). The signal was detected by enhanced chemiluminescence (ECL) (Amersham) following the manufacturer's instructions. The images shown in the figures are representative of three independent experiments.

### 2.12 RNA Extraction and RT–PCR

Total RNA was isolated from the cells via TRIzol reagent (Molecular Research Center, No. TR118) according to the manufacturer's protocol. The RNA was reverse transcribed into cDNA via an iScript cDNA Synthesis Kit (Bio‐Rad, No. 1708891). Real‐time PCR was performed in 96‐well plates on a CFX96 Touch Real‐Time PCR Detection System (Bio‐Rad) using iTaq Universal SYBR Green Supermix (Bio‐Rad, No. 1725121). The targeted mRNA expression in each sample was normalised to that of the endogenous control (18S). The relative mRNA expression was quantified via the 2^−ΔΔ*Ct*^ method. Each experiment was repeated at least three times. The following primers were used for mouse genes to detect corresponding mRNAs via RT–PCR: IL‐6 FQP, 5′‐CTGCAAGAGACTTCCATCCAG‐3′; IL‐6 RQP, 5′‐AGTGGTATAGACAGGTCTGTTGG‐3′; IL‐10 FQP, 5′‐CTTACTGACTGGCATGAGGATCA‐3′; IL10 RQP, 5′‐GCAGCTCTAGGAGCATGTGG‐3′; 18S FQP, 5′‐GTAACCCGTTGAACCCCATT‐3′; and 18S RQP, 5′‐CCATCCAATCGGTAGTAGCG‐3′.

The following primers were used for human genes: CCL5 FQP, 5′‐TGCAGAGGATCAAGACAGCA‐3′; CCL5 RQP, 5′‐GAGCACTTGCCACTGGTGTA‐3′; CXCL10 FQP, 5′‐TCTGAATCCAGAATCGAAGG‐3′; and CXCL10 RQP, 5′‐CTCTGTGTGGTCCATCCTTG‐3′.

### 2.13 Animal Study

Female wild‐type C57BL/6J mice were obtained from Guangdong Medical Laboratory Animal Center. All the subcutaneous tumour experiments were performed with 7‐ to 12‐week‐old female mice maintained in the conventional mouse facility. The mice were randomly grouped, and a total of 5 × 10^5^ LLC‐siNC or LLC‐siCx43 cells in 50 μL of PBS were injected into the mice to establish tumours. When the tumour volume reached 50–100 mm^3^, the mice were treated with PD‐1 antibody alone or in combination with 2′,3′‐cGAMP. A total of 200 μg PD‐1 antibody in 100 μL of PBS was intraperitoneally injected into the mice. The mice were intratumorally injected with a total of 2 μg 2′,3′‐cGAMP in 20 μL of PBS. These treatments were repeated three times at 7‐day intervals. The tumour size was measured with a digital calliper and the tumour volume was calculated via the formula *V* = (*L* × *W* ^2^)/2 and expressed as mm^3^, where *V* is the tumour volume, *L* is the length of the tumour (longer diameter) and *W* is the width of the tumour (shorter diameter).

### 2.14 Immunohistochemistry Analysis

A tissue microarray of human lung adenocarcinoma and paired adjacent normal tissues (HLugA060PG03) was purchased from Shanghai Outdo Biotech Company (Shanghai, China). The study was approved by the Ethics Committee of Shanghai Outdo Biotech Company. Immunohistochemical analysis of Cx43, STING, CD8, CD68 expression, and the tumour microenvironment was performed by staining with anti‐Cx43 (Servicebio, No. GB11234), anti‐STING (STARTER, No. S0B0084), anti‐CD68 (Servicebio, No. GB113150) and anti‐CD8 (Servicebio, No. GB12068) antibodies via the labelled streptavidin–biotin method. Briefly, paraffin‐embedded tissue sections were dewaxed with xylene and then hydrated with graded concentrations of ethanol. The sections were placed in 3% hydrogen peroxide solution and incubated at room temperature for 25 min in the dark to block endogenous peroxidase activity. After washing, nonspecific binding sites were blocked by incubating the slides with 3% BSA (Servicebio, No. GC305010) for 30 min at room temperature, followed by incubation with primary antibody overnight at 4°C. The sections were washed and incubated with the appropriate secondary antibody conjugated to HRP at room temperature for 50 min and stained with DAB staining reagent (Servicebio, No. G1212). After counterstaining with haematoxylin and dehydration, the sections were mounted and imaged via a Leica microscope. The tissue microarrays were evaluated by two independent pathologists, who were blinded to the experiment. Based on the HALO platform of Indica labs (USA), a positive area analysis of the immunohistochemical scan results was performed. The histochemistry score (*H*‐score) was used to evaluate the results of immunohistochemistry staining, which comprehensively evaluated the proportion of positive cells and staining intensity.

### 2.15 Statistical Analysis

Statistics were analysed either by 1‐way ANOVA or Student's *t*‐test where applicable. Statistical analysis was performed via the SPSS (16.0; SPSS, Inc.) and GraphPad Prism (Prism 8.0; GraphPad Software Inc.) packages. The error bars represent the means ± SDs. A *p* value < 0.05 was considered significant. All the quantitative experiments were repeated in at least three independent tests.

## 3 Results

### 3.1 Cx43 Was Associated With Increased CD8 ^+^ T‐Cell Infiltration and a Better PFS in Human Lung Cancer

**FIGURE 1:**
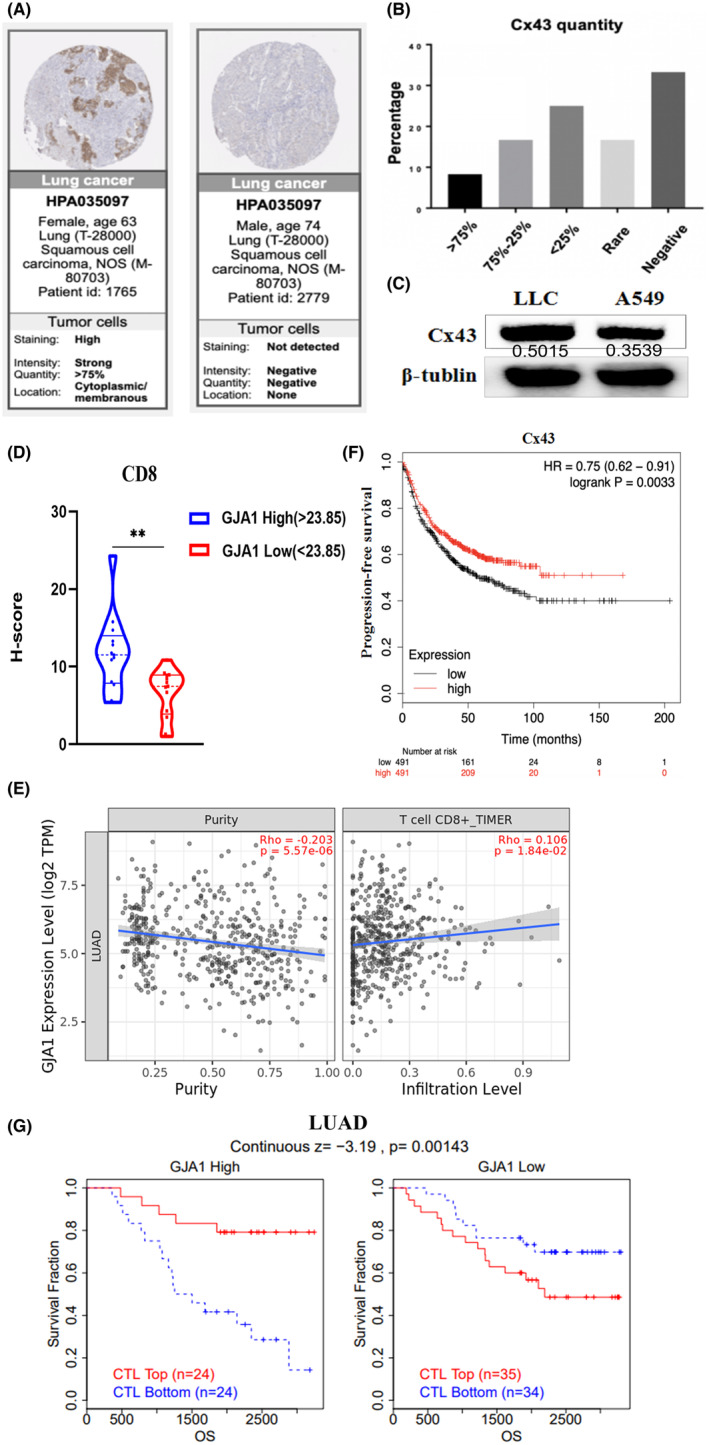
Cx43 was associated with increased CD8^+^ T‐cell infiltration and a better PFS in human lung cancer. (A, B) The protein expression level of Cx43 in the tumor tissues of lung cancer patients was determined via the Human Protein Atlas (www.proteinalas.org/) database. (C) Western blot analysis of Cx43 expression in LLC and A549 cells. (D) Immunohistochemical analysis of CD8 *H*‐scores difference between Cx43‐high (*H*‐score > 23.85) and Cx43‐low (*H*‐score < 23.85) groups. *n* = 15. (E) Relevance analysis among GJA1 and CD8^+^ T cell. (F) The correlation between Cx43 expression and PFS in lung cancer patients was analyzed via Kaplan–Meier estimates via the KM plotter online database (www.kmplot.com). (G) The TIDE database analyzes the connection between GJA1and CTL. **p < 0.01.

The Human Protein Atlas (www.proteinalas.org/) revealed that the protein expression of Cx43 in the tumour tissues of most lung cancer patients was reduced or absent, with only 9% of patients having a Cx43 expression intensity greater than 75% (Figure 1A,B). Cx43 expression varied among different lung cancer cells (Figure 1C). To investigate the relationship between Cx43 and T cell infiltration in human lung cancer, 30 lung adenocarcinoma patients' tumour tissues were collected. Based on the IHC scores of each tumour tissue, patients with lung cancer were divided into Cx43‐high (*H*‐score > 23.85) and Cx43‐low (*H*‐score < 23.85) groups according to the median value of Cx43 expression, and the *H*‐scores of CD8 were compared between these two groups. CD8 was the significant symbols of CD8 cytotoxic T lymphocytes (CTLs). The data indicated that high expression of Cx43 in human lung adenocarcinoma tissues have higher CD8 expression (Figure 1D). These results showed that Cx43 was positively correlated with CD8 expression in human lung adenocarcinoma. To further evaluate the effect of Cx43 on the tumour immune microenvironment, we utilised TIMER2.0 to investigate the correlation between GJA1 expression and infiltration level of CD8^+^ T cells in LUAD. The analysis demonstrated a significant positive correlation between GJA1 expression and infiltration level of CD8^+^ T cells (Figure 1E).

A positive correlation between Cx43 expression and progression‐free survival (PFS) in lung cancer patients was detected via the Kaplan–Meier plotter tool (kmplot.com) (Figure 1F). These results indicate that Cx43 might be associated with better PFS in lung cancer patients. Moreover, TIDE database was used to reveal the correlation between GJA1 and CTL dysfunction, which promotes adaptive immune resistance, leading to tumour immune escape. We found that GJA1 was negatively correlated with the degree of CTL dysfunction. High levels of GJA1 expression leads to increased CTL infiltration, which enhances antitumor immunity and prolongs survival in these patients. However, these patients with low GJA1 expression reversed this result (Figure 1G).

### 3.2 Knockdown of Cx43 Decreased cGAMP‐Mediated Anti‐PD‐1‐Induced Cell Death In Vitro and In Vivo

**FIGURE 2:**
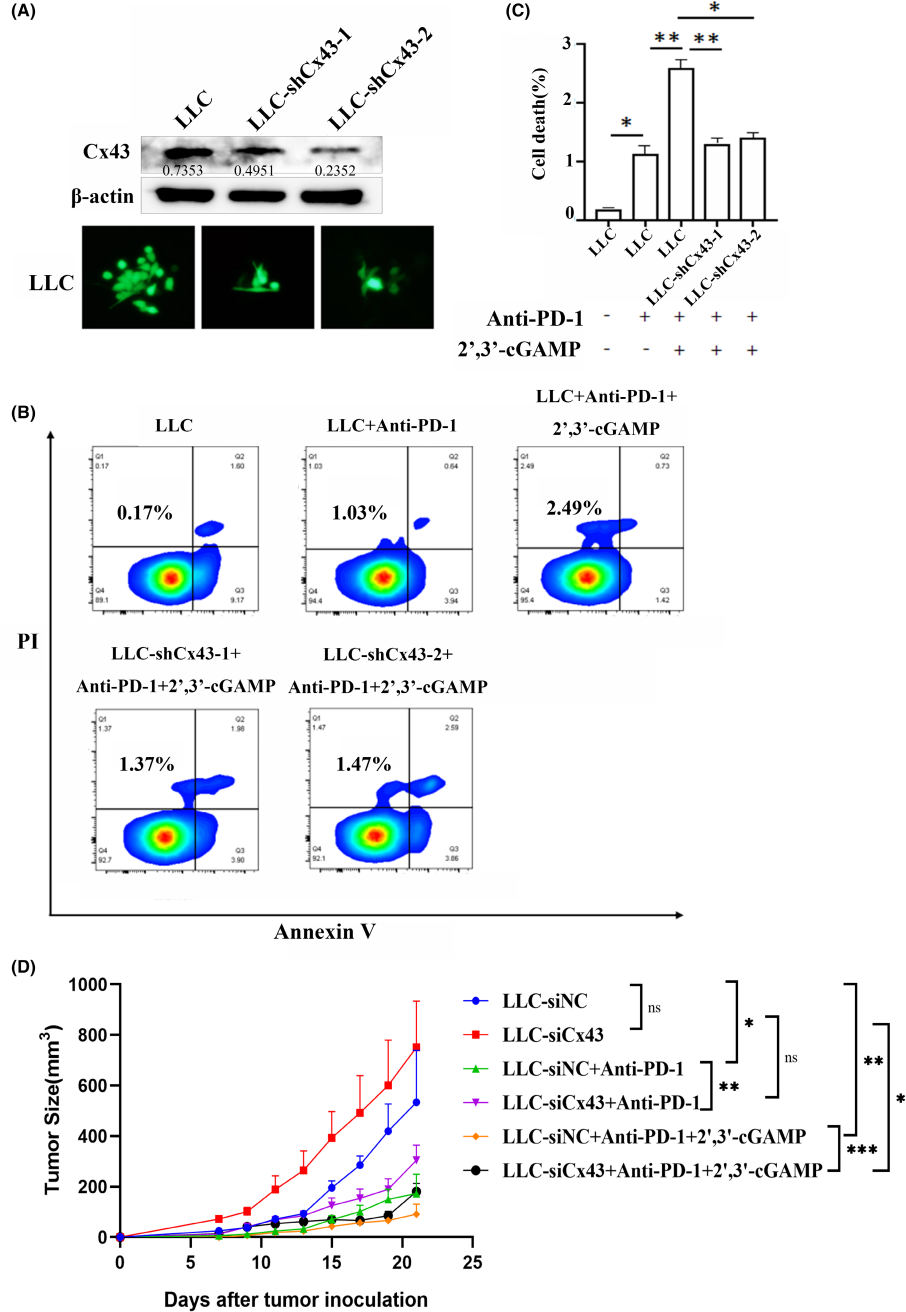
Knockdown of Cx43 decreased cGAMP‐mediated anti‐PD‐1‐induced cell death in vitro and in vivo. (A) The expression of Cx43 in LLC, LLC‐shCx43‐1 and LLC‐shCx43‐2 cells was assessed via western blotting. (B) Apoptotic cells were labeled via Annexin V/PI staining and quantitatively assessed via flow cytometry. (C) Each experiment was performed at least three times. (D) C57BL/6J mice (*n* = 6–8 pre group) were injected with 5 × 10^5^ LLC‐siNC or LLC‐siCx43 cells, followed by treatments with anti‐PD‐1 or combined cGAMP. Tumor volumes were measured on indicated dates. Data are shown as mean ± SEM. ns, not significant, *p < 0.05, **p < 0.01, ***p < 0.001.

To determine whether Cx43 contributes to anti‐PD‐1‐induced cell death in vitro, we knocked down Cx43 in the LLC cell line via shRNA. Compared with control cells, both LLC‐shCx43‐1 and LLC‐shCx43‐2 cells presented dramatic decreases in Cx43 levels and reduced GJIC function (Figure 2A). As shown by the flow cytometry results, anti‐PD‐1 antibody alone induced 1.03% cell death after 48 h of treatment and pretreatment with cGAMP increased the percentage of anti‐PD‐1 antibody‐induced cell death to 2.49%, as indicated by the number of PI^+^ Annexin V^−^ cells (Figure 2B,C). Interestingly, when 2′,3′‐cGAMP was added to LLC‐shCx43 cell lines prior to anti‐PD‐1 therapy, the number of PI^+^ Annexin V^−^ cells was decreased by approximately 50% compared with that in LLC cells. To further verify the effect of Cx43 on cGAMP and anti‐PD‐1 on LLC cells in vivo LLC cells with Cx43 silenced by siRNA transfection (Figure S1A) were used to construct a subcutaneous tumour mouse model. By comparing the tumour size of mice in different treatment groups, we found that silencing Cx43 did not affect the tumour growth of mice without anti‐PD‐1 treatment, while silencing Cx43 blocked the inhibitory effect of anti‐PD‐1 on tumour. When combined with 2′,3′‐cGAMP, the inhibitory effect of anti‐PD‐1 on tumour was restored (Figure 2D). The results indicated that Cx43 can affect the death of tumour cells, improve the sensitivity of tumour cells to anti‐PD‐1 and inhibit tumour growth. Both in vitro and in vivo experiments showed that cGAMP transfer and anti‐PD‐1 sensitivity were reduced by Cx43 knockdown.

### 3.3 Inhibition of Cx43 in LLC Cells Attenuated T‐Cell Antitumor Immunity via Decreasing cGAMP Transfer to Macrophages

**FIGURE 3:**
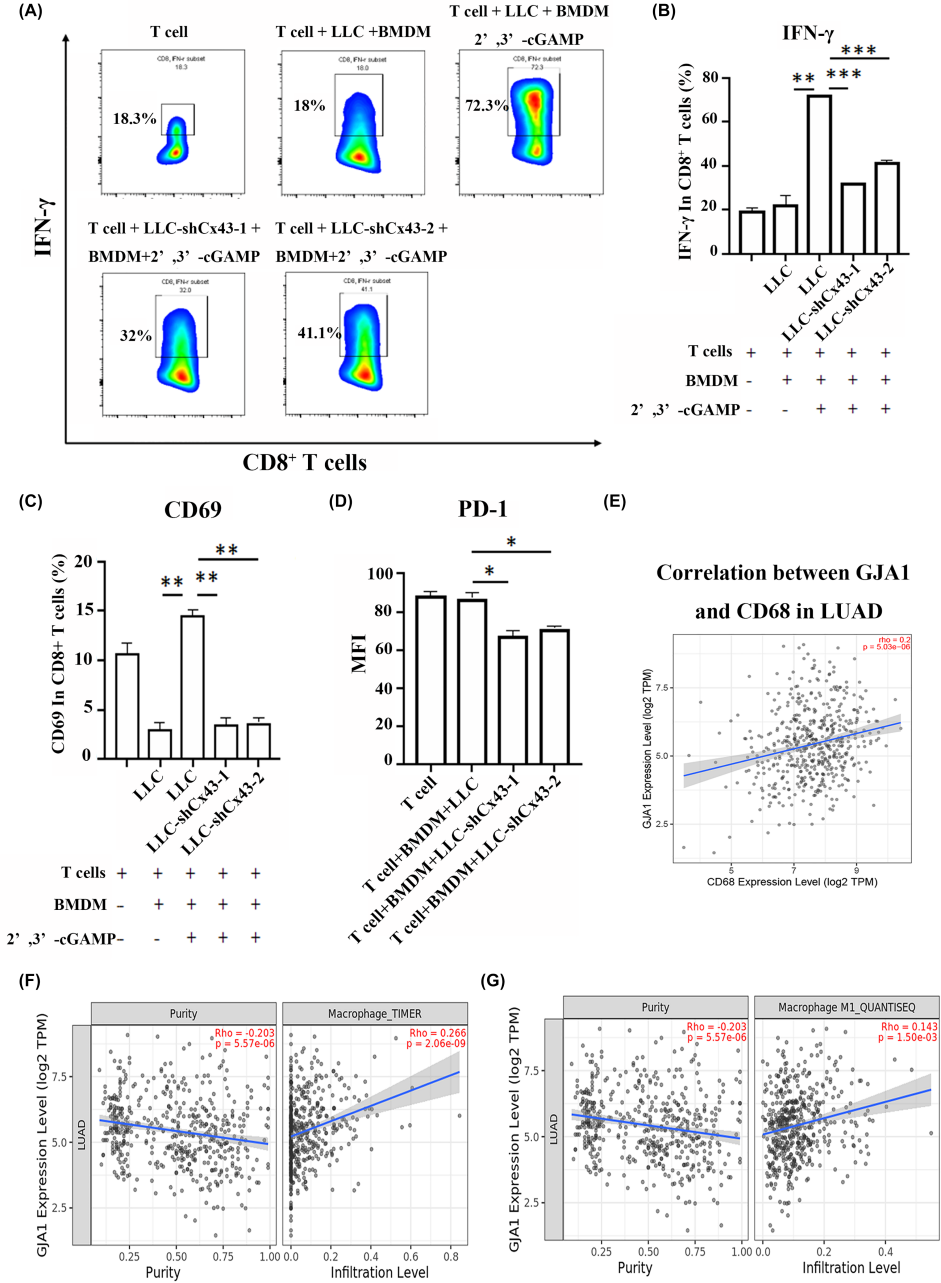
Inhibition of Cx43 in LLC cells attenuated T‐cell antitumor immunity via cGAMP transfer to macrophages. (A) Representative flow cytometric analysis of IFN‐γ+ expression in CD8^+^ T cells. (B–D) Quantification of the expression of IFN‐γ, CD69 and PD‐1 in CD8^+^ T cells. Each experiment was performed at least three times. (E) Correlation between GJA1 and CD68 in LUAD. *n* = 515. (F, G) Relevance analysis among GJA1 and Macrophage, Macrophage M1. *p < 0.05, **p < 0.01, ***p < 0.001.

To test whether cGAMP contributes to T‐cell antitumor immunity, we cocultured LLC cells with BMDMs for 24 h in the presence or absence of 2′,3′‐cGAMP and then added splenocytes at a 5:1 ratio for another 72 h. CD8^+^ T‐cell activity, IFN‐γ secretion, and CD69 and PD‐1 expression were analysed. LLC or BMDM alone did not affect T‐cell activity. However, pretreatment of LLC cells with 2′,3′‐cGAMP increased the number of CD8^+^ IFN‐γ^+^ T cells approximately threefold, as did CD69 expression, indicating that cGAMP contributes significantly to T‐cell antitumor immunity (Figure 3A–C). We further evaluated the effect of Cx43 in LLC cells on cGAMP‐mediated T‐cell antitumor immunity by coculturing BMDMs with 2′,3′‐cGAMP‐pretreated LLC or LLC‐shCx43 cells for 24 h and then adding splenocytes at a 5:1 ratio for another 72 h. Interestingly, knocking down Cx43 in LLC cells significantly reduced CD8^+^ IFN‐γ^+^ T cells and CD69 expression by approximately 50%, indicating that Cx43 mediated cGAMP transfer from LLC cells to macrophages, thus activating T cells. In addition, PD‐1 expression was significantly downregulated when Cx43 was knocked down in LLC cells (Figure 3D). Thus, Cx43/GJ mediates communication between tumour cells and macrophages via the transfer of cGAMP, thereby resulting in enhanced antitumor T‐cell immunity.

We further investigated the relationship between GJA1 and CD68 expression levels within the tumour microenvironment, with CD68 being a well‐established marker for macrophages. The correlation analysis revealed a significant positive association between GJA1 and CD68 expression (Figure 3E). The TIMER2.0 analysis demonstrated a significant positive correlation between GJA1 expression and infiltration level of total macrophages, as well as M1 macrophages (Figure 3F,G), suggesting that GJA1 may be involved in macrophage‐mediated immune responses in lung carcinoma.

### 3.4 Inhibition of Cx43 in LLC Cells Promoted TAM Polarisation

**FIGURE 4:**
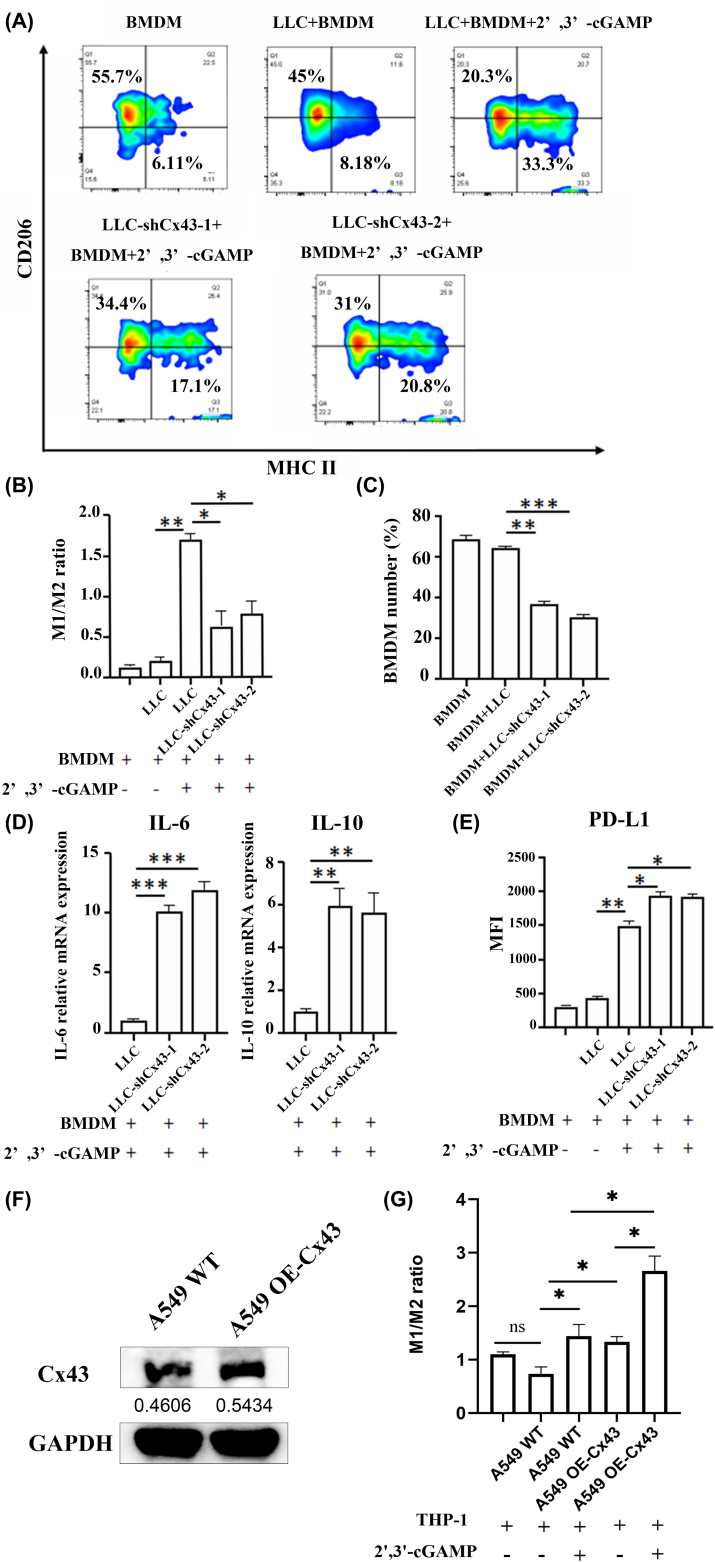
Inhibition of Cx43 in LLC cells induced macrophage polarisation to TAMs. (A) Representative flow cytometric analysis of CD206 and MHC II expression in BMDMs co‐cultured with LLC or LLC‐shCx43 cells with or without cGAMP. (B, C) M1/M2 ratio and number of BMDMs co‐cultured with LLC or LLC‐shCx43 cells. (D) Relative mRNA expression of IL‐6 and IL‐10 in BMDMs co‐cultured with LLC or LLC shCx43 cells in the presence of cGAMP, as determined via RT–PCR. (E) Quantification of the expression of PD‐L1 in BMDMs from the LLC group, LLC‐shCx43 group and control group with or without cGAMP. Each experiment was performed at least three times. (F) Western blotting verified the efficiency of Cx43 overexpression in A549. (G) M1/M2 ratio of THP‐1 co‐cultured with A549 WT or A549 OE‐Cx43 cells with or without cGAMP. ns, not significant, *p < 0.05, **p < 0.01, ***p < 0.001.

To assess the role of cGAMP in macrophages, we cocultured BMDMs with LLC cells with or without 2′,3′‐cGAMP. Neither the M1/M2 ratio nor the total BMDM number changed with the presence of LLC cells. However, adding 2′,3′‐cGAMP prior to LLC cells significantly increased the M1/M2 ratio and total BMDM number, indicating that cGAMP indeed promoted BMDM to the antitumor type (Figure 4A–C). We then cocultured LLC or LLC‐shCx43 cells with BMDMs in the presence of 2′,3′‐cGAMP. The M1/M2 ratio and total number of BMDMs were significantly decreased when Cx43 was knocked down. In addition, increased mRNA levels of IL‐6 and IL‐10, as well as increased expression of PD‐L1, were observed in BMDMs cocultured with LLC‐shCx43 cells compared with LLC cells, indicating that knocking down Cx43 in tumour cells induced macrophages towards the protumor type (Figure 4D,E). We obtained the same results in Cx43 silenced LLC cell line transfected with si‐Cx43 (Figure S1B–D).

Next, we cocultured THP‐1 and A549 WT or A549 OE‐Cx43 cells (Figure 4F) with or without 2′,3′‐cGAMP. Accordingly, both Cx43 overexpression and 2′,3′‐cGAMP treatment increased the M1/M2 ratio, and the addition of cGAMP further increased the M1/M2 ratio (Figure 4G, Figure S1E).

### 3.5 Inhibition of Cx43 in LLC Cells Led to Reduced STING Expression in Macrophages

**FIGURE 5:**
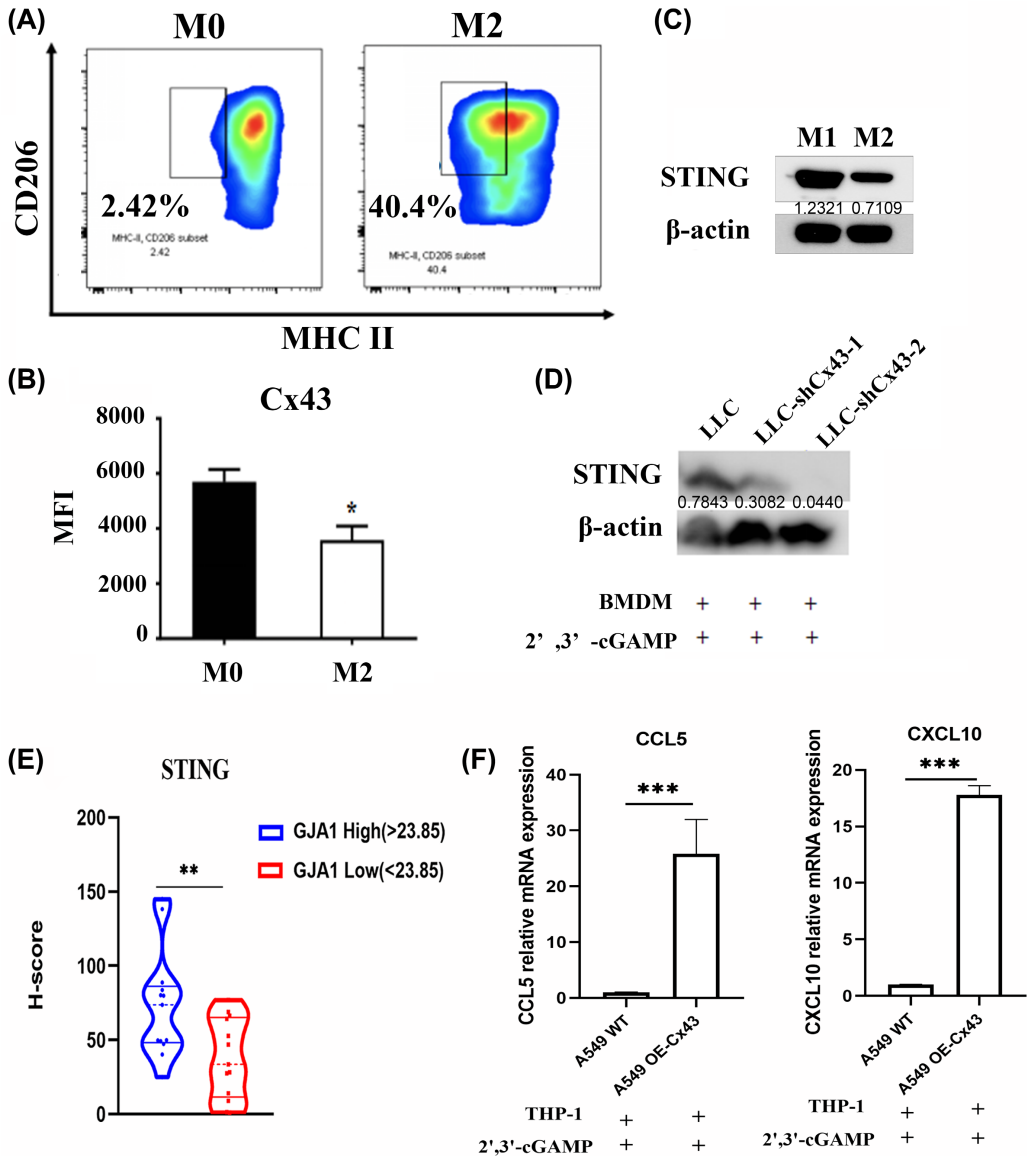
Inhibition of Cx43 in LLC cells decreased STING expression in BMDMs. (A) Representative flow cytometric analysis of CD206 and MHC II expression after IL‐4 stimulation in BMDMs for 48 h. (B) Quantification of the expression of Cx43 in M0 and M2 BMDMs. (C) The expression of STING in M0 and M2 BMDMs was assessed via western blotting. (D) The expression of STING in BMDMs co‐cultured with LLC or LLC‐shCx43 cells pretreated with cGAMP was determined via western blotting. Each experiment was performed at least three times. (E) Immunohistochemical analysis of STING *H*‐scores difference between Cx43‐high (*H*‐score > 23.85) and Cx43‐low (*H*‐score < 23.85) groups. *n* = 15. (F) Relative mRNA expression of CCL5 and CXCL10 in THP‐1 cocultured with A549 WT or A549 OE‐Cx43 cells in the presence of cGAMP, as determined via RT–PCR. *p < 0.05, **p < 0.01, ***p < 0.001.

To further investigate how LLC cells induce M2 macrophages, BMDMs were polarised into M2‐type macrophages with the stimulus IL‐4. After the addition of IL‐4 to BMDMs for 48 h, flow cytometric analysis revealed a significant increase in the expression of the M2‐type marker CD206 compared with the M0‐type marker, indicating that the BMDMs were successfully induced from the M0 type to the M2 type (Figure 5A). Furthermore, we analysed Cx43 expression in M0 and M2 macrophages and observed that Cx43 expression was significantly lower in M2 macrophages than in M0 macrophages, which further verified that knocking down Cx43 in LLC cells led to attenuated T cell antitumor immunity (Figure 5B). 2′,3′‐cGAMP is considered a STING agonist and has been proven to have antitumor effects. STING was expressed at lower levels in M2 macrophages than in M1 macrophages (Figure 5C). To evaluate the effect of Cx43 on STING expression, we pretreated LLC or LLC‐shCx43 cells with 2′,3′‐cGAMP and cocultured them with BMDMs. STING expression in BMDMs was significantly lower when co‐cultured with LLC‐shCx43 cells than when co‐cultured with LLC cells, indicating that STING in macrophages was deactivated when Cx43 was blocked in tumour cells, which further resulted in a decreased antitumor effect on T cells (Figure 5D).

According to the IHC scores of tumour tissue, the *H*‐scores of STING were compared between Cx43‐high and Cx43‐low groups. These data indicated that high expression of Cx43 in human lung adenocarcinoma tissues have higher STING expression (Figure 5E). Activation of cGAS–STING signal can increase the production of downstream chemokines CCL5 and CXCL10 [37], which were markers of STING pathway activation. We examined the mRNA expression of CCL5 and CXCL10 after coculture of THP‐1 and A549 WT or A549 OE‐Cx43 cells in the presence of 2′,3′‐cGAMP. After overexpression of Cx43, the mRNA expression of CCL5 and CXCL10 increased significantly (Figure 5F), suggesting that Cx43 activated the STING pathway.

## 4 Discussion

The KEYNOTE‐024 study showed that in metastatic NSCLC patients with at least 50% PD‐L1‐positive tumour cells, compared with platinum‐based chemotherapy, pembrolizumab prolonged PFS by 4.3 months and increased the 6‐month overall survival (OS) rate from 72.4% to 80.2% [2]. However, this study did not involve patients with negative PD‐L1 expression. The KEYNOTE‐189 study reported that the combination of pembrolizumab and platinum drugs prolonged the PFS of patients with metastatic NSCLC by 3.7 months and improved the 10.5‐month OS from 49.4% to 69.2% compared with placebo and platinum drugs [38], but the greatest benefit of this regimen was still in patients with a PD‐L1‐positive rate greater than 50%. Even with pembrolizumab as preoperative neoadjuvant therapy, the response rate is only 45% [39]. In addition, the PACIFIC study reported that durvalumab prolonged the OS and PFS of stage III NSCLC patients after chemoradiotherapy, whereas 46% of the patients still did not respond to the regimen [40]. Data mining analysis of data from the Human Protein Atlas database and KM plotter online database revealed that high Cx43 expression was highly correlated with improved PFS. The expression of Cx43 may serve as a predictive indicator of clinical outcomes in cancer treatment.

This study revealed that Cx43 in LLC cells increased sensitivity to anti‐PD‐1 therapy. When 2′,3′‐cGAMP was added to LLC‐shCx43 or LLC cells prior to anti‐PD‐1 treatment, LLC‐shCx43 cell death decreased by approximately 50% compared with that in LLC cells (Figure 2B,C), indicating that cGAMP plays an important role in anti‐PD‐1 therapy and that Cx43 knockdown decreased cGAMP transfer to BMDMs. 2′,3′‐cGAMP is considered a STING agonist that can bind to STING to activate downstream immune responses [26]. Studies have shown that cytoplasmic dsDNA produced by tumour cells can be delivered to macrophages, activate the cGAS–STING pathway, activate T cells and increase the sensitivity of these cells to ICIs [27]. In addition, other studies have combined AXL inhibitors, which inhibit dendritic cells, increase the number of TCF1^+^ CD8^+^ T cells in the tumour microenvironment and restore the PD‐1 blockade sensitivity of STING‐inhibited LKB1‐mutant NSCLC [41]. Thus, we investigated whether Cx43 could overcome ICI resistance via the cGAS–STING pathway. We cocultured LLC or LLC‐shCx43 cells with BMDMs in the presence of 2′,3′‐cGAMP and found that when Cx43 was knocked down in LLC cells, the mRNA levels of IL‐6 and IL‐10, as well as the expression of PD‐L1, were increased in BMDMs, indicating that the inhibition of LLC Cx43 induced macrophage polarisation to TAMs (Figure 4D,E). Furthermore, we analysed Cx43 expression in M0‐ and M2‐type macrophages and observed that Cx43 expression was significantly downregulated in M2‐type macrophages compared with M0‐type macrophages (Figure 5B). More importantly, we found that inhibiting Cx43 in LLC cells reduced the expression of STING in macrophages. These data suggest that the knockdown of Cx43 in LLC cells leads to ICI resistance by negatively regulating the cGAS–STING pathway in macrophages. The possible mechanisms of the Cx43‐mediated cGAS–STING interaction between lung cancer cells and TAMs remain to be further investigated.

In summary, our results demonstrated that Cx43 could enhance antitumor T‐cell immunity by mediating communication between tumour cells and macrophages via the transfer of cGAMP in LLC cell models. The inhibition of Cx43 in lung cancer cells can induce macrophage polarisation into TAMs and lead to ICI resistance by negatively regulating the cGAS–STING pathway in macrophages. Therefore, Cx43/GJ‐mediated signal transmission between lung cancer cells and macrophages provides new insights for increasing the sensitivity of lung cancer cells to immunotherapy.

## Author Contributions

**Yuan Zhang:** conceptualization (equal), data curation (equal), formal analysis (equal), funding acquisition (equal), investigation (equal), methodology (equal), project administration (equal), supervision (equal), validation (equal), visualization (equal), writing – original draft (equal), writing – review and editing (equal). **Yu Gao:** conceptualization (equal), data curation (equal), formal analysis (equal), funding acquisition (equal), investigation (equal), methodology (equal), project administration (equal). **Jie Wang:** conceptualization (equal), data curation (equal), formal analysis (equal), investigation (equal), methodology (equal), supervision (equal), validation (equal), visualization (equal), writing – original draft (equal), writing – review and editing (equal). **Xiaoming Tan:** conceptualization (equal), data curation (equal), formal analysis (equal), investigation (equal), methodology (equal), project administration (equal), supervision (equal), validation (equal), visualization (equal), writing – original draft (equal), writing – review and editing (equal). **Zihua Deng:** data curation (equal), formal analysis (equal), investigation (equal). **Jingjing Zhou:** data curation (equal), formal analysis (equal), investigation (equal). **Guoqing Luo:** conceptualization (equal), data curation (equal), formal analysis (equal), funding acquisition (equal), investigation (equal), methodology (equal), project administration (equal), resources (equal), supervision (equal), validation (equal), visualization (equal), writing – original draft (equal), writing – review and editing (equal). **Yusheng Liang:** conceptualization (equal), data curation (equal), formal analysis (equal), funding acquisition (equal), investigation (equal). **Weize Yu:** conceptualization (equal), data curation (equal), formal analysis (equal), funding acquisition (equal), investigation (equal). **Zu Ye:** conceptualization (equal), data curation (equal), formal analysis (equal), funding acquisition (equal), investigation (equal).

## Conflicts of Interest

The authors declare no conflicts of interest.

## Supporting information


Figure S1.

## Data Availability

Data sharing is not applicable to this article as no new data were created or analyzed in this study.
