# Supplementary material for: Connexin 43 Deficiency Confers Resistance to Immunotherapy in Lung Cancer via Inhibition of the Cyclic GMP‐AMP Synthase–Stimulator of Interferon Genes Pathway
Source: J Cell Mol Med. 2024 Nov 26;28(22):e70211. doi: 10.1111/jcmm.70211 (PMC11598135; doi:10.1111/jcmm.70211)
Supplement: Supplementary file 1 — Figure S1. [file JCMM-28-e70211-s001.zip › Supplemental Figure Legend.docx]

Figure legends:

Supplemental Figure 1. Cx43 affects the polarization of macrophages.

(A). The expression of Cx43 protein in LLC cells transfected with si-GJA1 was detected by western blotting. (B). Representative flow cytometric analysis of CD206 and MHC II expression in BMDMs co-cultured with LLC-siNC or LLC-siCx43 cells with or without cGAMP. (C). M1/M2 ratio of BMDMs cocultured with LLC-siNC or LLC-siCx43 cells. (D). Relative mRNA expression of IL-6 and IL-10 in BMDMs co-cultured with LLC-siNC or LLC-siCx43 cells in the presence of cGAMP, as determined via RT‒PCR. (E). Representative flow cytometric analysis of CD206 and CD86 expression in THP-1 cocultured with A549 WT or A549 OE-Cx43 cells with or without cGAMP. **P < 0.01, ***P < 0.001
