# Supplementary figures and images for: Connexin 43 Deficiency Confers Resistance to Immunotherapy in Lung Cancer via Inhibition of the Cyclic GMP‐AMP Synthase–Stimulator of Interferon Genes Pathway
Source: J Cell Mol Med. 2024 Nov 26;28(22):e70211. doi: 10.1111/jcmm.70211 (PMC11598135; doi:10.1111/jcmm.70211)

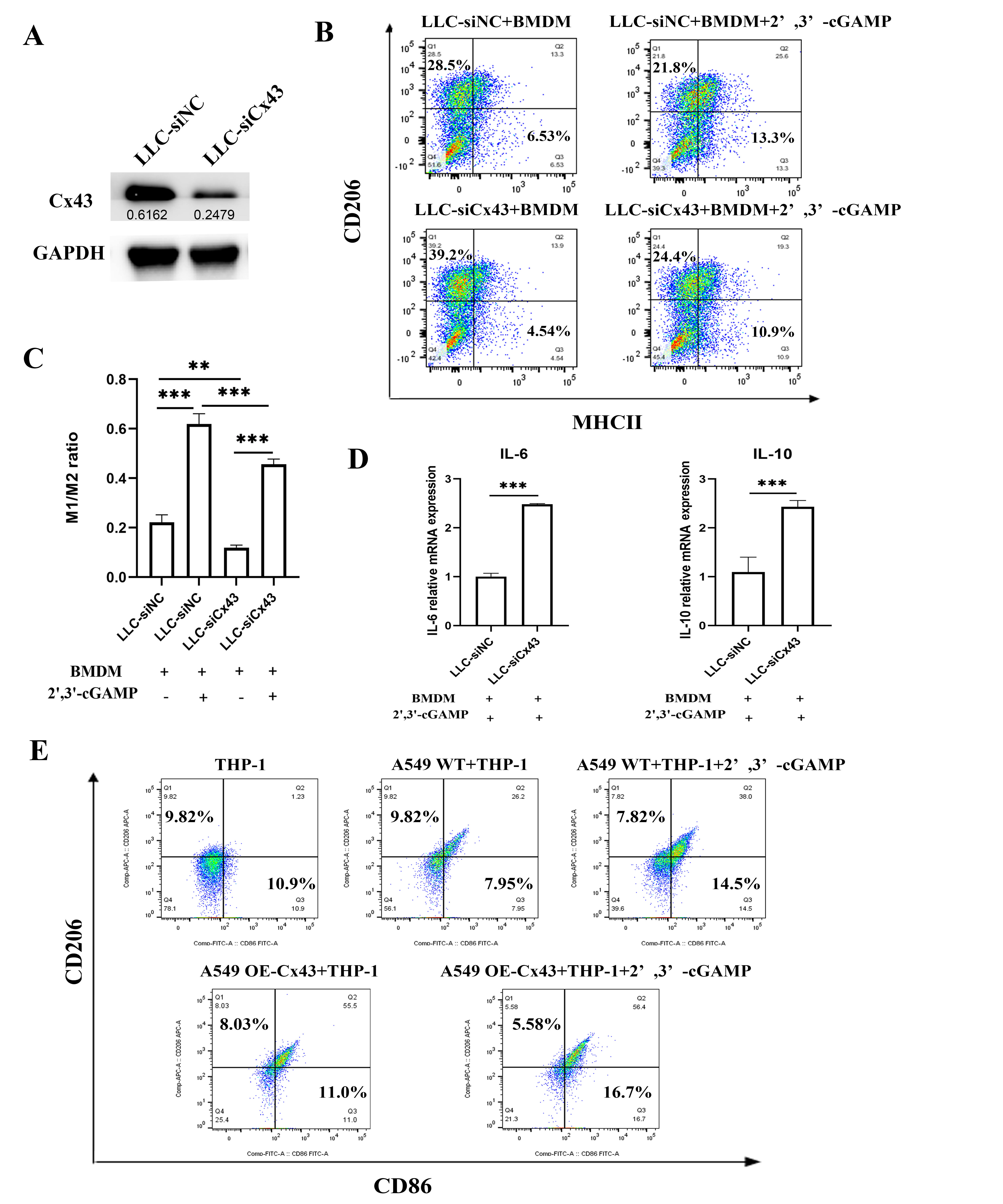

Supplement: Supplementary file 1 — Figure S1. [file JCMM-28-e70211-s001.zip › Supplemental Figure 1.tif]
